# Cross cultural adaptation and analysis of psychometric properties of Sinhala version of Menopause Rating Scale

**DOI:** 10.1186/s12955-018-0977-9

**Published:** 2018-08-06

**Authors:** Nirmala Rathnayake, Janaka Lenora, Gayani Alwis, Sarath Lekamwasam

**Affiliations:** 1 Department of Nursing, Faculty of Allied Health Sciences, University of Ruhuna, Galle, Sri Lanka; 2 Department of Physiology, Faculty of Medicine, University of Ruhuna, Galle, Sri Lanka; 3 Department of Anatomy, Faculty of Medicine, University of Ruhuna, Galle, Sri Lanka; 4 Population Health Research Center, Department of Medicine, Faculty of Medicine, University of Ruhuna, Galle, Sri Lanka

**Keywords:** Cross cultural adaptation, Menopause rating scale, Psychometric properties, Reliability, Sinhala version, Validity

## Abstract

**Background:**

Menopause Rating Scale (MRS) evaluates eleven menopausal symptoms and health related quality of life (HRQOL) of postmenopausal women under three subscales. In this study we attempted cross cultural adaptation and evaluation of psychometric properties of a Sinhala translation of MRS.

**Methods:**

Sinhala version of MRS was adapted following standard methodology; forward and backward translations, review by an expert group, focus group discussion (FGD) and pre-testing. It was self-administered among randomly selected healthy, Sinhalese, community-dwelling 166 postmenopausal women (aged; median = 56.5, IQR, 53.0–59.0 years) along with the Short Form 36 (SF-36) survey questionnaire. MRS was re-administered among a subsample (*n* = 80) after two weeks of first administration. Psychometric properties; reliability and validity were evaluated.

**Results:**

In Sinhala version of MRS, both internal consistency (Cronbach’s alpha coefficient = 0.79) and test retest reliability (intra class correlation / ICC = 0.86, 95%CI = 0.82–0.91, *p* < 0.001 and Pearson correlation / *r* = 0.93) were high. Factor analysis (FA) with Principal Component Analysis (PCA) extracted three factors explaining 59.82% cumulative variance with few exceptions from the original version. In the item-subscale correlation analysis items showed stronger correlations within their own subscale score (r range between 0.56–0.84) than with other subscales scores and subscales' scores showed strong correlations with the overall MRS score (r range between 0.70–0.86) indicating strong convergent validity. Mean (SD) symptom severities of each item were significantly different between symptomatic and asymptomatic women (*p* < 0.05) emphasizing good discriminant validity. The overall MRS and SF-36 scores correlated significantly (Pearson correlation: − 0.52, *p* < 0.01 and Kendall’s tau-b: − 0.39, p < 0.01) ensuring strong criterion validity.

**Conclusions:**

The Sinhala version of MRS we adapted is an informative tool with high reliability and validity and this tool can be used to evaluate the menopausal symptoms and HRQOL in postmenopausal women conversant in Sinhala.

**Electronic supplementary material:**

The online version of this article (10.1186/s12955-018-0977-9) contains supplementary material, which is available to authorized users.

## Background

Assessment of Health Related Quality of Life (HRQOL) of postmenopausal women has become an important aspect in contemporary health care as the HRQOL of middle aged women is influenced by adverse physical and psychological changes after menopause [1, 2]. Menopause Rating Scale (MRS) is a widely used tool to assess both the HRQOL in postmenopausal women and the severity of menopausal symptoms [3].

MRS, originally designed by Hauser et al. [4] assesses eleven menopause-related symptoms and their severity in interviewer-administered manner. In 1996 Potthoff et al., evaluated its psychometric properties using a group of postmenopausal women [5] with self-administration of the tool. Potthoff et al. categorized the eleven items into three independent dimensions or subscales namely somato-vegetative symptoms, psychological symptoms and urogenital symptoms [5] based on the Factor Analysis (FA).

MRS has been subsequently translated to 27 different languages. However, it has not been translated to Sinhala language. Apart from the evaluation of the severity of menopausal symptoms and HRQOL, MRS can be used as a screening tool to identify those who need higher level of care for severe menopausal symptoms [6]. Researchers and clinicians in Sri Lanka should have an access to a validated tool to evaluate the severity of menopausal symptoms among local subjects in order to facilitate further research and improve the HRQOL of postmenopausal women. Therefore, this study was designed for cross cultural adaptation and evaluation of psychometric properties of a Sinhala translation of MRS to evaluate its suitability as a tool for assessing severity of menopausal symptoms and HRQOL of postmenopausal women.

## Methods

### Study design, setting and participants

This validation study was conducted in the Bope-Poddala Medical Officer of Health (MOH) area; University field training area in Galle district in Southern Sri Lanka. The study participants in both cross cultural adaptation and psychometric validation were healthy community dwelling postmenopausal women who attained menopause naturally with intact uterus. Those who had severe medical and surgical conditions, mental illnesses, disabilities and impairments of musculoskeletal system, endocrine disorders (diabetes, thyroid…etc) were excluded from the study. Women on hormone replacement therapy (HRT) were also excluded. Postmenopausal status was determined based on self-reported menstrual history using the classification of Stages of Reproductive Aging Workshop (STRAW) [7] which is cessation of menstruation within the previous 12 months after last menstruation.

### Cross cultural adaptation of MRS to Sinhala language

The standard guidelines described by Beaton et al. [8] were followed for cross cultural adaptation of Sinhala version of MRS. The original English version was translated (forward translation) to Sinhala language by two independent health professionals; a medical officer and a nurse, who were fluent in both Sinhala and English languages. One was informed about the purpose of translation while the other was not informed. Then the items were consolidated in to a single questionnaire to maximize the clarity of items by the investigators together and synthesized a common translation. The synthesized translation was back translated to English language (backward translation) by two independent health professionals, fluent in both Sinhala and English languages, to assess the comparability with the original version and to make sure that there were no gross inconsistencies or conceptual errors. A group of experts (physician, gynecologist, physiologist, anatomist and nursing academic and forward and backward translators) independently reviewed all the versions of MRS and decided a pre-final Sinhala version. They also ensured semantic equivalence, idiomatic equivalence, experiential equivalence and conceptual equivalence with the original version and finally the content validity was ensured while producing a pre-final version. The pre-final version ensured by the expert committee was further assessed for clarity, understandability and naturalness of items in a focus group discussion (FGD) with 10 postmenopausal women with similar inclusion criteria. The final version (Additional file 1) was pretested among 30 postmenopausal women with above inclusion criteria selected from a different geographical area in the Galle district and the face validity was ensured.

### Administration of Sinhala version of MRS

The newly adopted Sinhala version of MRS and previously validated Sinhala version of Short Form (SF-36) survey [9, 10] were self-administered among 166 postmenopausal women selected from the university training area to evaluate its psychometric properties.

These women were selected by multistage cluster sampling technique. Out of the 18 Public Health Midwife divisions (the smallest community health unit) of Bope-Poddala MOH area, three were selected randomly. Houses and the female household aged beyond 40 years were identified using the electoral registers of the respective areas, obtained from Grama Niladari (the local community administrative authority) of each division. Women who got odd numbers when the houses were arranged in a single list were invited for the study (excluding the women who had exclusion criteria) until the required sample size achieved.

For the sample size calculation, 15 respondents in to one variable ratio was used [11]. Then sample size was calculated by multiplying the number of variables in the instrument (11 items) with 15 (in order to achieve a best sample) and it was 165. After adding 10% to compensate with the non-respondents and incomplete questionnaires, the final sample size was calculated as 181. Even though we invited 181 women, only 174 women participated and only 166 postmenopausal women completed all the items of questionnaires (response rate was 91.7%).

MRS was re-administered to a subgroup of postmenopausal women (*n* = 80) randomly selected from the main group, two weeks after the first administration.

For each item in the MRS, women were asked to indicate whether they had experienced such symptom or problem within the previous month. The severity of the symptoms was assessed by 5 point Likert scale; from 0 (no symptom at all) to 4 (very severe). The total severity (ranged from the minimum of 0 to the maximum of 44) was determined by adding the scores of the three subscales.

The SF-36 survey is a multi-purpose; short-form health survey consisted of 36 items, which provide a subjective estimation of the individual’s functional state and HRQOL in two main dimensions (physical and psychological) [9] and has been used in the validation of MRS to ensure the criterion validity [12]. In SF-36 each dimension is given a score ranging from 0 to 100 using the original coding algorithm and higher values indicate higher HRQOL [9].

### Statistical analyses

The socio-demographic characteristics of the study participants in psychometric validation step are presented as frequencies (percentages) or median (IQR).

The test-retest reliability of the MRS was examined by intra-class correlation coefficient (ICC) and Pearson correlation coefficient (r) comparing the overall MRS scores and subscale scores [13–15] of two consecutive administrations of MRS. Internal consistency was assessed with Cronbach’s alpha for overall MRS and subscales and a Cronbach’s alpha equal or more than 0.5 was regarded acceptable based on the results of the previous MRS validation studies [16]. Convergent validity was tested by item-subscale correlation considering higher correlation of each item with their respective subscale.

Construct validity was evaluated by FA performed with PCA while keeping Varimax with Keiser normalization as rotation method to determine whether the latent item structure mirrored the three domains specified in the instrument construction. The Kaiser-Meyer-Olkin (KMO) and Bartlett’s test of sphericity statistics were analyzed and correlation matrix was observed to determine whether FA was appropriate for the data, mainly for sampling adequacy assessment (KMO > 0.7), multicollinearity assessment (many coefficients in correlation matrix should be 0.3 and above), Bartlett’s Test of Sphericity should reach statistical significance (*p* < 0.001) and Commonalities Coefficients should be high (> 0.6) [17].The number of extracted components was determined by the Scree plot, percentage of variance explained by each component, number of Eigen values over one (Kaiser-Guttman rule), and consideration of prior psychometric MRS analyses. Items were considered representative of a component if their individual item loading was ≥0.40 and in the cross-loading items, the factor, which had higher loading value, was taken as the respective factor [17].

Discriminant validity was measured by comparison of menopausal symptomatic and asymptomatic women for each item [18], compared with independent sample t test. Asymptomatic and symptomatic women in this study were based on the severity of menopausal symptoms; absence of symptoms and mild symptoms in five-point Likert scale (0–1) were considered as asymptomatic and other severities (2–4 in Likert scale) were taken as symptomatic women.

Criterion validity was calculated with Pearson correlation coefficients and Kendall’s tau-b value between the overall scores of MRS and SF 36 survey and separately physical and psychological dimension scores were also observed [13–15].

Statistical analysis was performed with SPSS 20.0 version (IBM statistics, Inc., Chicago) and *P* value < 0.05 was considered as statistically significant. Correlation values between 0.10 and 0.29 were considered weak, between 0.30 and 0.49 were considered moderate, and between 0.50 and 1.00 were considered strong [17].

### Ethical considerations

Permission from the original authors of MRS was sought before the commencement of the validation process. Ethical clearance for the study was obtained from the Ethical Review Committee, Faculty of Medicine, University of Ruhuna and written informed consent was obtained from each participant.

## Results

### Characteristics of the participants

Age of the subjects ranged from 45 to 60 (median, IQR = 56.5, 53.0–59.0) years while age at menopause and duration since menopause ranged from 40 to 56 (median, IQR = 49.0, 45.0–51.0) years and 2 to 20 (median, IQR = 6.5, 3.0–10.5) years, respectively (Table 1). Sociodemographic characteristics of the participated women are presented in Table 1.

**Table 1:** Sociodemographic characteristics of the study participants (*n* = 166)

Characteristics	Sub category	Frequency (%) or Median (IQR)
Age (years)		56.5 (53.0–59.0)
Age at menopause (years)		49.0 (45.0–51.0)
Duration since menopause (years)		6.5 (3.0–10.5)
Employment status	Employed	40(24.0)
	Non employed	126(76.0)
Civil status	Married	120(75.9)
	Single/widowed/divorced	47(24.1)
Living companion	With husband and children	111(66.8)
	With Husband/ Children	30(18.0)
	Alone or living with others	25(15.2)
Education status	Primary education	46(27.7)
	Secondary education	72(43.3)
	Upper secondary education, degree or diploma	48(29.0)
Monthly income	Below 20,000 SLR	132(79.5)
	Above 20,000 SLR	34(20.5)

LKR = Sri Lankan rupees (150LKR = 1USD)

Living with others include; parents, siblings, friends or other relatives

Majority of women had joint and muscular complaints, physical and mental exhaustion and sleep disturbances. Sexual problems, vaginal dryness and heart discomfort were less prevalent (Table 2).

**Table 2:** Prevalence of menopausal symptoms among study participants (*n* = 166)

Item no.	Prevalence of menopausal symptoms	Asymptomatic women (n, %)	Symptomatic women (n, %)
1	Hot flushes, sweating (episodes of sweating)	75(45.2)	91(54.8)
2	Heart discomfort (unusual awareness of heartbeat, heart skipping, heart racing, tightness	104(67.2)	62(37.3)
3	Sleep problems (difficulty in falling asleep, difficulty in sleeping through, waking up early)	69(41.6)	97(58.4)
4	Depressive mood (feeling down, sad, on the verge of tears, lack of drive, mood swings)	80(48.2)	86(51.8)
5	Irritability (feeling nervous, inner tension, feeling aggressive)	77(46.4)	89(53.6)
6	Anxiety (inner restlessness, feeling panicky)	80(48.2)	86(51.8)
7	Physical and mental exhaustion (general decrease in performance, impaired memory, decrease in concentration, forgetfulness)	45(27.1)	121(72.9)
8	Sexual problems (change in sexual desire, in sexual activity and satisfaction)	115(69.3)	51(30.7)
9	Bladder problems (difficulty in urinating, increased need to urinate, bladder incontinence	97(58.4)	69(41.6)
10	Dryness of vagina (sensation of dryness or burning in the vagina, difficulty with sexual intercourse)	111(66.9)	55(33.1)
11	Joint and muscular discomfort (pain in the joints, rheumatoid complaints)	20(12.0)	146(88.0)

Asymptomatic and symptomatic women in this study were based on their severity of menopausal symptoms; absence of symptoms and mild symptoms in five-point Likert scale (0–1) were considered as asymptomatic and other severities (2–4 in Likert scale)

In the MRS, median (IQR) values of somato-vegetative, psychological, uro-genital and overall scores were 4.0(2.0–6.0), 5.0 (3.0–7.0), 1.0 (0.0–3.0) and 10.0 (6.0–16.0), respectively.

### Psychometric properties of Sinhala version of MRS

#### Reliability and internal consistency

In the test-retest reliability assessment, the Pearson correlation coefficients for the overall MRS score was 0.93 (*p* < 0.01) while ICC was 0.86 (95% CI = 0.87–0.97). Subscale scores also showed high test-retest reliability; somato-vegetative symptoms: *r* = 0.92, ICC = 0.95, 95%CI = 0.86–0.94, psychological symptoms: *r* = 0.94, ICC = 0.93, 95%CI = 0.89–0.95 and urogenital symptoms: *r* = 0.77, ICC = 0.77, 95%CI = 0.67–0.85. MRS showed a high internal consistency with global Cronbach’s alpha of 0.74, and three subscales; somato-vegetative symptoms, psychological symptoms and urogenital symptoms; 0.50, 0.76 and 0.62, respectively.

#### Convergent validity

In the item-subscale correlation analysis items correlated with their own subscale better (r ranged between 0.56–0.84, *p* < 0.01) than with the other subscales, indicating high convergence of items in their respective subscale (Table 3). Further, each subscales showed strong correlations with the overall MRS (r ranged between 0.70–0.86, *p* < 0.01) (data not shown).

**Table 3:** Item-subscale correlation analysis of MRS (11 items)

Item no.	Item	Correlation coefficient			
		Somato-vegetative symptoms	Psychological symptoms	Uro-genital symptoms	Overall MRS score
1	Hot flushes, sweating	**0.63**	0.33	0.27	0.52
2	Heart discomfort	**0.56**	0.39	0.39	0.55
3	Sleep problems	**0.63**	0.30	0.17	0.50
4	Depressive mood	0.40	**0.83**	0.38	0.69
5	Irritability	0.43	**0.84**	0.42	0.72
6	Anxiety	0.35	**0.78**	0.30	062
7	Physical and mental exhaustion	0.49	**0.62**	0.35	0.62
8	Sexual problems	0.28	0.34	**0.80**	0.62
9	Bladder problems	0.41	0.36	**0.64**	0.54
10	Dryness of vagina	0.23	0.37	**0.83**	0.55
11	Joint and muscular discomfort	**0.68**	0.40	0.26	0.57

Items are presented in short forms

Pearson correlation was significant at < 0.01 level. Corresponding items of the subscales are shown bold

#### Construct validity

**Fig. 1:**
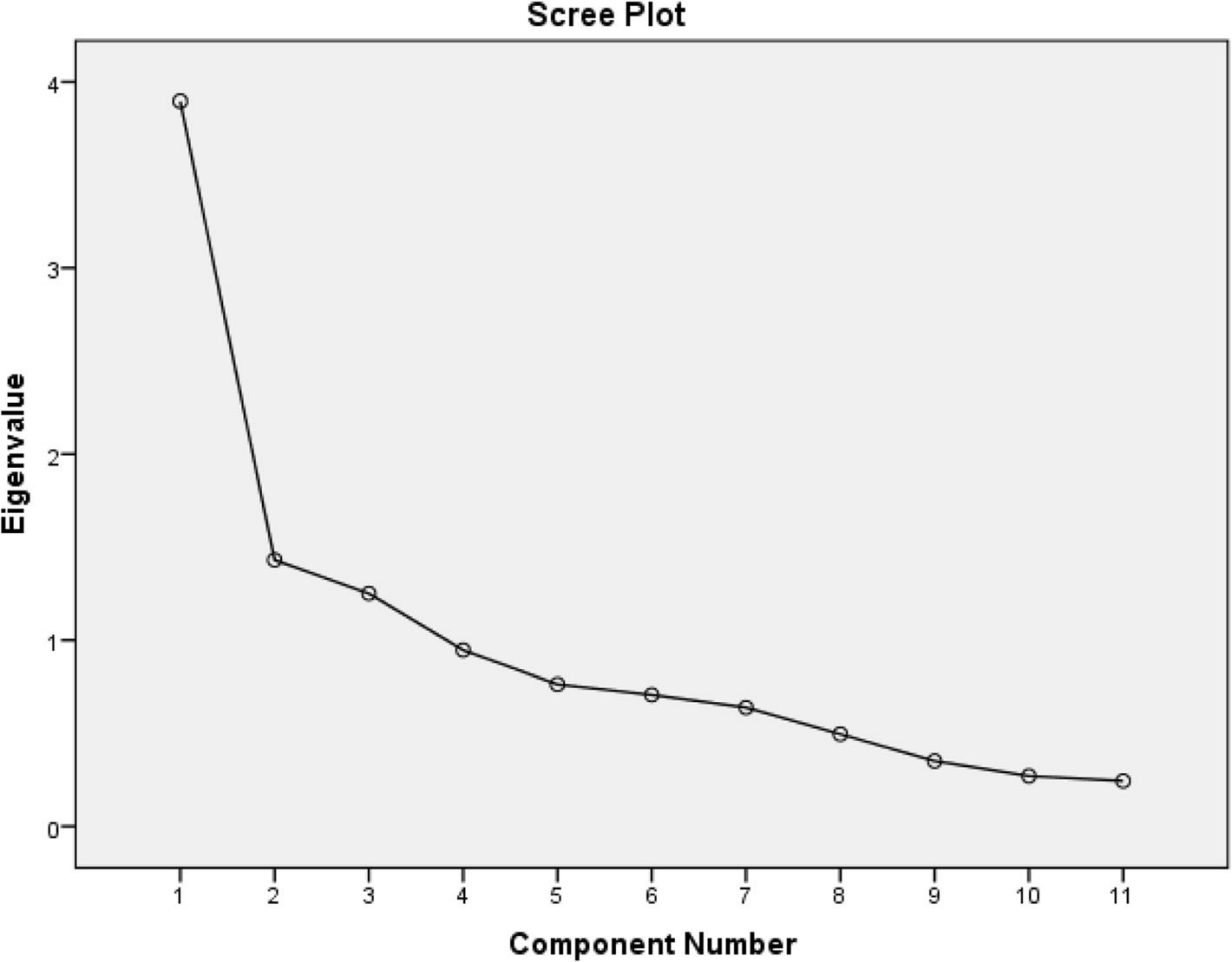
Screen plot of factor extracting in MRS with PCAᅟ

Correlation matrix revealed the presence of many coefficients> 0.3 (data not shown) while the KMO value was 0.78, and Bartlett’s Test of Sphericity reached statistical significance (p < 0.01) supporting the factorability of the correlation matrix. PCA revealed the presence of three factors with Eigen value exceeding 1, explaining cumulative variance of 59.82% with varimax rotation in the rotated component matrix (data not shown) and Scree plot (Fig. 1).

Items 4–6 which correspond to psychological symptoms saturated in to a single factor (factor 1) without cross loading items. It was purely named as “psychological problems” as in the original scale. Item 2 (heart discomfort), 8 (sexual problems) and 10 (vaginal dryness) saturated in to a single factor (factor 2) which is a combination of items in somato-vegetative symptoms and urogenital symptoms with a cross loading item (item 1; hot flushes/sweating). Therefore, it was named as “somato-sexual problems”. Item 3 (sleep problems), item 7 (physical and mental exhaustion), item 9 (bladder problems) and item 11 (joint and muscular complaints) saturated together in factor 3 and therefore, it was named as “physical problems”. Because item 1 cross loaded in factor 2 and 3 with higher loading in factor 3, it was considered as an item which should be in factor 3 (Table 4).

**Table 4:** Three factors extracted by the factor analysis with Varimax rotation

Subscale (As original scale)	Item	Item	Component (As FA suggested in this study)		
			1 (Psychological)	2 (Somato-sexual)	3 (Physical)
Somato-vegetative	1	Hot flushes, sweating		*0.40*	*0.56*
	2	Heart discomfort		*0.52*	
	3	Sleep disturbances			*0.56*
Psychological	4	Depressive mood	*0.80*		
	5	Irritability	*0.77*		
	6	Anxiety	*0.88*		
	7	Physical and mental exhaustion			*0.71*
Uro-genital	8	Sexual problems		*0.83*	
	9	Bladder problems			*0.62*
	10	Dryness of vagina		*0.86*	
Somato-vegetative	11	Joint and muscular discomfort			*0.60*

Items are presented in short forms, All coefficients are significant at *p*<0.01

Extraction Method: Principal Component Analysis, Rotation Method: Varimax with Kaiser Normalization

Factor 1 was named as “psychological”, Factor 2 was named as “somato-sexual” and Factor 3 was named as “physical” considering the nature of saturated items in this analysis

#### Discriminant validity

Mean (SD) scores of items based on the severity scale were significantly different (*p* < 0.01) between the asymptomatic and symptomatic women confirming the discriminant validity (Table 5).

**Table 5:** Comparison of symptomatic and asymptomatic women of menopausal symptoms (*n* = 166)

Item no.	Item	Comparison of asymptomatic and symptomatic women			
		Asymptomatic women Mean (SD)	Symptomatic women Mean (SD)	T value	*p* value
1	Hot flushes, sweating	0.34(0.47)	2.60(0.79)	−22.61	< 0.01
2	Heart discomfort	0.30(0.46)	2.53(0.51)	−18.66	< 0.01
3	Sleep problems	0.30(0.45)	2.71(0.83)	−23.97	< 0.01
4	Depressive mood	0.33(0.47)	2.39(0.64)	−22.50	< 0.01
5	Irritability	0.40(0.49)	2.32(0.62)	−19.83	< 0.01
6	Anxiety	0.38(0.48)	2.37(0.58)	−21.11	< 0.01
7	Physical and mental exhaustion	0.48(0.50)	2.54(0.71)	−21.69	< 0.01
8	Sexual problems	0.21(0.41)	2.55(0.75)	−21.11	< 0.01
9	Bladder problems	0.27(0.44)	2.58(0.79)	−22.30	< 0.01
10	Dryness of vagina	0.23(0.42)	2.68(0.83)	−21.63	< 0.01
11	Joint and muscular discomfort	0.61(0.49)	3.06(0.83)	−19.60	< 0.01

Items are presented as short forms

Asymptomatic and symptomatic women in this study were based on their severity of menopausal symptoms; absence of symptoms and mild symptoms in five-point Likert scale (0–1) were considered as asymptomatic and other severities (2–4 in Likert scale)

Comparison of two groups was performed with independent sample t test

#### Criterion validity

The Pearson correlation coefficient and Kendall’s tau-b observed between the overall MRS score and overall SF-36 survey scores and between the respective factors after FA of MRS and SF 36 survey (physical symptoms score of MRS with physical dimension of SF 36 survey and psychological symptoms score of MRS with psychological dimension of SF 36 survey) were high that indicate strong criterion validity of the MRS (Table 6).

**Table 6:** Correlation between Sinhala version of MRS and SF 36 survey scores

MRS		SF 36 survey					
		Overall score		Physical dimension		Psychological dimension	
		r	K-tb	r	K-tb	r	K-tb
Overall score	r	−0.53**					
	K-tb		−0.39**				
Physical symptom score	r			−0.47**			
	K-tb				−0.33**		
Psychological symptom score	r					−0.45**	
	K-tb						−0.37**

**Correlation is significant at < 0.01 level

r = Pearson correlation, K-tb = Kendall’s tau b

## Discussion

In the current study, we evaluated the psychometric properties of the Sinhala version of the MRS. We observed that the Sinhala version of MRS has sound psychometric properties that are concordant with previous MRS validation studies [3, 16, 19, 20].

Furthermore, we observed a satisfactory firmness and repeatability with an acceptable test-retest reliability and internal consistency which are compatible with the previous studies [3, 16, 19, 20]. Our analysis showed a three factor model accounting for 59.8% of the total variance which is almost identical as the first FA where three factor model accounted for 58.8% of the total variance [5]. Even though we found a three factor model in our analysis a few exceptions were observed. Three items belonging to psychological symptoms saturated together while one psychological symptom (physical and mental exhaustion) saturated with somatic and urinary symptoms. One somato-vegetative symptom (heart discomfort) saturated with sexual symptoms. The variation in factor extraction observed in this study could be due to several reasons. Primarily, the prevalence of menopausal symptoms has a regional variation. In our study sample, women mostly complained of musculoskeletal symptoms but complains related to sexual problems and heart discomforts were less frequent. Similar observations have been made in previous studies conducted in Sri Lanka [2, 21]. As the prevalence of heart discomfort, dryness of vagina and sexual problems was comparatively low, those three items saturated together. As, hot flushes, sleep problems, physical and mental exhaustion, bladder problems and musculoskeletal problems were more prevalent, they saturated in one factor. Further, women might get misclassified in symptoms related to physical and mental exhaustion as they may be unable to distinguish them from psychological impact.

However, results similar to the current study have been observed in previous validation studies. For an example, musculoskeletal problems have got loaded in both somato-vegetative and urogenital subscales in a study done in the USA [16] while sleep disturbances in both somato-vegetative and psychological subscales in a collaborative study conducted in Latin America; Spain, Mexico and Brazil [16]. Further, sleep disorders saturated with psychological symptoms while bladder problems saturated with somato-vegetative symptoms in a Chinese study [19]. Furthermore, two factor model in which both somato-vegetative and psychological symptoms saturated as a single factor occurred in a validation study done in Czech Republic [3].

We observed an inverse correlation between the total and subscale scores of MRS and SF 36 indicating that higher scores of MRS are associated with lower SF-36 scores and lower HRQOL among these women. Similar results have been observed in previous studies too [3, 12].The concordance of the findings of different studies on psychometric properties could partly be due to comparable sample sizes, age range and sampling characteristics used in our study and previous studies conducted in Czech Republic and China [3, 19]. Therefore, MRS has shown its validity in different geographical locations when correct methodology is used. However, the studies with methodological deviations [16] have shown variations in psychometric properties compared to ours and above cited studies [3, 19].

We found that the MRS is user friendly, time efficient as it requires only five minutes to complete and sufficiently sensitive to measure the HRQOL of postmenopausal women. The observed sound psychometric properties of this Sinhala version of MRS indicate its potential to be used in both community and clinical settings for the assessment of menopause related symptoms and HRQOL in women conversant in Sinhala language, especially in the remote and rural areas where the health care facilities are less accessible.This study has a few limitations to mention. Due to the unavailability of a menopause-specific QOL assessment tool in Sri Lanka we had to use general SF-36 questionnaire to evaluate the criterion validity of the scale. And we performed only the exploratory FA in this analysis due to the lack of software to perform the confirmatory FA. Furthermore, our findings cannot be generalized to postmenopausal women who speak languages other than Sinhala. Therefore, we encourage further studies in other locations with postmenopausal women with different educational and social background to assess the reliability of these results including the confirmatory FA.

## Conclusion

This study indicates that the Sinhala version of the MRS we adapted is a time efficient, practical, and informative tool with sound validity and reliability. Therefore, it can be used to assess menopause related symptoms severity and HRQOL among Sinhala speaking postmenopausal women in Sri Lanka and could be used as a screening tool to identify those women in need of referral to higher level of care for severe menopausal symptoms.

## Additional file

Additional file 1: “Sinhala version of Menopause Rating Scale”. NB – This manuscript was produced from the findings generated in the PhD degree of NR. (PDF 268 kb)

## Abbreviations

CI: Confidence interval
FA: Factor analysis
FGD: Focus group discussion
HRQOL: Health related quality of life
HRT: Hormone replacement therapy
ICC: Intra class correlation
KMO: Kaiser-Meyer-Olkin
MOH: Medical Officer of Health
MRS: Menopause Rating Scale
PCA: Principle component analysis
SD: Standard deviation
SF 36: Short form 36 survey
STRAW: Stages of reproductive aging workshop
USA: United States of America
