# Supplementary material for: Cross cultural adaptation and analysis of psychometric properties of Sinhala version of Menopause Rating Scale
Source: Health Qual Life Outcomes. 2018 Aug 6;16:161. doi: 10.1186/s12955-018-0977-9 (PMC6080547; doi:10.1186/s12955-018-0977-9)
Supplement: Supplementary file 1 — “Sinhala version of Menopause Rating Scale”. NB – This manuscript was produced from the findings generated in the PhD degree of NR. (PDF 268 kb) [file 12955_2018_977_MOESM1_ESM.pdf]

ආර්තවහරණ ඇගයීමේ දර්ශකය

නම .... වයස (අවුරුදු)..

පහත සඳහන් රෝග ලක්ෂණ පසුගිය සති හතරක (මාසයක) කාලය තුල ඔබට බලපෑ ආකාරය අදාළ කොටුවේ ලකුණු කරන්න. එම ලක්ෂණ ඔබ අත් නොවින්දේ නම් ‘‘නැත’’ යන කොටුවේ ලකුණු කරන්න.

රෝග ලක්ෂණය	නැත	සුළු වශයෙන්	මධ්‍යස්ථ වශයෙන්	දැඩි ලෙස	ඉතා දැඩි ලෙස
1. නිතර නිතර දහදිය දැමීම හෝ සිරුරේ දැවිල්ලක් හටගැනීම	☐	☐	☐	☐	☐
2. පපුවේ අපහුතාවයක් දැනීම (අසාමාන්‍ය වූ හදවත ස්පන්දනය වීම, හදගැස්ම අතරමග නැවතීම, හදවත වේගයෙන් ස්පන්දනය වනබව.... දැනීම, පපුව සිරවීමක් වැනි හැගීමක් ඇති වීම)	☐	☐	☐	☐	☐
3. නින්ද ආශ්‍රිත අපහසුතා (නිදමත ගතයක් නොදනිම, නින්ද නොයාම, දිගු නින්දක් ලබාගත නොහැකි වීම, වඩා වේලාසන අවදි වීම)	☐	☐	☐	☐	☐
4. කනස්සලු ස්වභාවය (දුක්බර හැගීම් ඇතිවීම, මානසික අසහනය හෝ අවපීඩනය, නිතර ඇඬෙන සුළු ස්වභාවය, උද්යෝගිමත් බව අඩුවීම, නිතර තරහ යාම)	☐	☐	☐	☐	☐
5. නොසන්සුන් භාවය (සිතේ කලබලකාරී බව, ආවේගශීලි බව, නිතර කැළඹෙන බව)	☐	☐	☐	☐	☐
6. සිතේ කාංසාව (ඇතුලාන්තයේ චිත්ත ආවේගවල නොසන්සුන් බව, අස්ථාන බියක් ඇතිවීම, අවිවේකි බවක් දැනීම)	☐	☐	☐	☐	☐
7. කායික හා මානසික විඩාව (සාමාන්‍ය ක්‍රියාශීලී බව අඩුවීම, මතක ශක්තිය අඩුවීම, යම් දෙයක් කෙරෙහි වූ අවධානය නිතර වෙනස් වීම, ධාරණ ශක්තිය අඩුවීම, නිතර අමතක වීම)	☐	☐	☐	☐	☐
8. ලිංගික ගැටළු (ලිංගික ක්‍රියාකාරකම් සඳහා ඇති කැමැත්ත වෙනස් වීම, ලිංගික ක්‍රියාකාරකම් සීමා කිරීම, ලිංගික ක්‍රියාකාරකම් වලින් ලැබෙන තෘප්තිය අඩු වීම)	☐	☐	☐	☐	☐
9. මුත්‍රාශය ආශ්‍රිත ගැටළු (මුත්‍රා පිට කිරීමේ අපහසුතා ඇතිවීම, මුත්‍රා පහ කිරීමට අවශ්‍ය වාර ගණන වැඩි වීම, මුත්‍රා පිට වීම පාලනය කල නොහැකි වීම, කරන හෝ කිවිසන විට නොදනී මුත්‍රා පිටවීම)	☐	☐	☐	☐	☐
10. යෝනි මාර්ගයේ වියලි බව (යෝනි මාර්ගයේ වියලි බව හෝ දැවිල්ලක් ඇතිවීම, ලිංගික සංසර්ගයේදී අපහසුතා ඇතිවීම)	☐	☐	☐	☐	☐
11. සංධි හා පේශි වල අපහසුතාවය (සංධි වල වේදනාවක් ඇතිවීම, රක්ත වාත ගැටළු නිතර උත්සන්න වීම)	☐	☐	☐	☐	☐
